# Commentary: Long-Term Exercise Reduces Formation of Tubular Aggregates and Promotes Maintenance of Ca^2+^ Entry Units in Aged Muscle

**DOI:** 10.3389/fphys.2021.663677

**Published:** 2021-04-01

**Authors:** Alexandra Salvi, André Maues De Paula, Nicolas Lévy, Shahram Attarian, Marc Bartoli

**Affiliations:** ^1^Aix Marseille Univ, INSERM, Marseille Medical Genetics, Marseille, France; ^2^Department of Anatomopathology, CHU La Timone, Marseille, France; ^3^Department of Medical Genetics, La Timone Children's Hospital, Marseille, France; ^4^Reference Center for Neuromuscular Disease and ALS, Marseille, France

**Keywords:** tubular aggregate myopathy, STIM 1, store operated Ca entry 2+, long-term exercise, genetics disease

## Introduction

We have read with great interest the study of Boncompagni et al. (2021) on the impact of long-term exercise in aged mice on tubular aggregates (TAs) formation and Ca^2+^ entry units (CEUs) maintenance. The authors showed a protective effect of long-term voluntary exercise on TA formation in a mouse model developing TAs in the course of aging. As stated by the authors, TAs presence in aged Human skeletal muscles has not been confirmed while it is a constant feature in tubular aggregate myopathy (TAM; MIM #160565 and #610277). TAM is a genetic disease characterized by progressive muscular weakness associated with specific histological features known as TAs. Mutations in STIM1 and ORAI1 encoding genes, the two regulators of store-operated calcium entry (SOCE) have been associated with TAM. The authors previously identified colocalization of these two proteins in CEUs, specific intracellular junctions between sarcoplasmic reticulum (SR) and T-tubules (Boncompagni et al., 2017). In their study, the authors showed an accumulation of these two proteins in aged mice muscles TAs.

We would like to comment on an interesting point in line with our previous study on TAM caused by *STIM1* mutations and, in particular, with the asymptomatic family described in our articles (De Paula et al., 2012; Böhm et al., 2013). Indeed, we are convinced that the present work in mice, associated with previous findings in TAM, will bring new insights in muscle diseases associated with tubular aggregates.

## Tubular Aggregates Myopathy: Asymptomatic and Symptomatic Families

In the first study on *STIM1* mutations associated with TAM (Böhm et al., 2013), we reported one asymptomatic family (Family 4). The proband, aged 30, presented with an elevated CK levels (27x normal levels) detected in a routine medical examination, while his younger brother, aged 24, and his father, aged 59, also presented elevated CK levels (5x and 9x normal levels respectively). None of them developed any muscular symptoms at the time of examination, although an electromyography analysis revealed a slight myopathic pattern for the proband. Further clinical exploration was proposed, and muscles biopsies revealed tubular aggregates, fiber size variation, type I fiber predominance, and type II fiber atrophy. Despite these histological alterations, no muscle weaknesses nor pain was evoked, even for the 59 years old father. Molecular genetics investigations revealed one missense mutation in *STIM1* c216C>G [p.(H72Q] in all three patients, affecting the canonical EF-hand in the luminal domain of STIM1 protein. At the time of the publication, we proposed two explanations for the absence of symptoms. First, the specific mutation [p.(H72Q)] by itself being correlated with a particularly mild clinical form; second, the patients' military duties with strong and regular physical training, could be considered as protective from muscle wasting.

Since then, three other families with mutation leading to the same amino acid modification c.216C>A [p.(H72Q)] have been identified (Morin et al., 2019). All the reported patients present muscle weakness occurring during childhood for one patient and during adulthood for the others. Histological analysis revealed classical TAM features together with TAs, fiber size variability and type I fiber predominance observed in muscle biopsies. Some patients also present fibers with centrally localized nuclei and/or vacuoles. These findings indicate that this amino acid mutation has a convincing causative effect on SOCE dysregulation leading to muscular weakness.

## Ca^2+^ Dysregulation and Remodeling of the SR

*In vitro* studies showed that mutation in STIM1 EF-hand lead to a constitutive activation, an abnormally excessive SOCE influx (Böhm et al., 2013, 2014) and probably to a cytosolic Ca^2+^ overload in cells. Tubular aggregates originate from the SR and previous studies suggest that their formation involved an altered proteostasis with aggregation of misfolded membrane protein leading to its remodeling (Schiaffino, 2012). The SR is the main Ca^2+^ stores in skeletal muscle.

Tubular aggregates originates from whole SR and with presence of different SR proteins in mice (Chevessier et al., 2004) and in human (Chevessier et al., 2005). Among these proteins, calsequestrin and SERCA are particularly increased in TAs (Boncompagni et al., 2012). On the one hand, the increase in SERCA protein suggest an increase in Ca^2+^ entry in SR. On the other hand, calsequestrin increase will decrease free Ca^2+^ concentration, despite an elevated Ca^2+^ entry.

In this study, the authors showed, an accumulation of STIM1 and ORAI1 proteins in TAs of aged muscle mice. These two proteins are responsible for SOCE influx.

Together the increased calsequestrin and SERCA proteins with the remodeling of the SR, in particular the formation of TAs through the elongation and increase of internal volume, would allow virtual control of Ca^2+^ overload.

## Discussion

While the precise and complete mechanism remains to be elucidated, according to these findings we suggest that tubular aggregates originate from an altered Ca^2+^ handling and that their presence is an attempt from the muscle fibers to control Ca^2+^ overload. Multiple events are associated with TAs development with SR enlargement, an increased in calsequestrin and SERCA and a trapping of SOCE proteins. Eventually, these mechanisms may be synergic or sequential to avoid Ca^2+^ overload with reducing SOCE proteins availability and a decrease in free Ca^2+^ concentration within the SR.

As Boncompagni and colleagues have shown in this study, long-term exercise decreases the number of TAs observed on muscle fibers from trained old mice, with a restoration of fatigue resistance and extracellular Ca^2+^ dependance through preservation of CEUs. Recently, Fodor and colleagues also indicated an improved Ca^2+^ homeostasis and force with training in aged mouse skeletal muscles (Fodor et al., 2020). We suggest that regular training prevents from Ca^2+^ dysregulation with a reduced cytosolic or SR Ca^2+^ overload, even with an enhanced SOCE influx. Indeed, Ca^2+^ dysregulation and TAs formation in skeletal muscles are found in asymptomatic (no muscular weakness) patients presenting with TAM.

We finally propose that physical activity may explain the absence of muscular weaknesses in this asymptomatic family with a maintained Ca^2+^ homeostasis. Thus, physical training may be considered as a therapeutic strategy to prevent the disease progression in TAM affected patients.

## Author Contributions

AS wrote and edited the manuscript. AM, NL, and SA were responsible for clinical investigations of the patients. MB conceived, directed the study and wrote the manuscript. All authors contributed to the article and approved the submitted version.

## Conflict of Interest

The authors declare that the research was conducted in the absence of any commercial or financial relationships that could be construed as a potential conflict of interest.
